# Mapping thalamocortical functional connectivity with large-scale brain networks in patients with first-episode psychosis

**DOI:** 10.1038/s41598-021-99170-7

**Published:** 2021-10-06

**Authors:** Yoo Bin Kwak, Kang Ik Kevin Cho, Wu Jeong Hwang, Ahra Kim, Minji Ha, Hyungyou Park, Junhee Lee, Tae Yong Lee, Minah Kim, Jun Soo Kwon

**Affiliations:** 1 Department of Brain and Cognitive Sciences, College of Natural Sciences, Seoul National University, Seoul, Republic of Korea; 2 Psychiatry Neuroimaging Laboratory, Department of Psychiatry, Harvard Medical School, Brigham and Women’s Hospital, Boston, USA; 3 Department of Psychiatry, Seoul National University College of Medicine, 101 Daehak-no, Chongno-gu, Seoul, 03080 Republic of Korea; 4 Department of Neuropsychiatry, Seoul National University Hospital, Seoul, Republic of Korea; 5 Department of Neuropsychiatry, Pusan National University Yangsan Hospital, Yangsan, Republic of Korea; 6 Institute of Human Behavioral Medicine, SNU-MRC, Seoul, Republic of Korea

**Keywords:** Neuroscience, Biomarkers

## Abstract

Abnormal thalamocortical networks involving specific thalamic nuclei have been implicated in schizophrenia pathophysiology. While comparable topography of anatomical and functional connectivity abnormalities has been reported in patients across illness stages, previous functional studies have been confined to anatomical pathways of thalamocortical networks. To address this issue, we incorporated large-scale brain network dynamics into examining thalamocortical functional connectivity. Forty patients with first-episode psychosis and forty healthy controls underwent T1-weighted and resting-state functional magnetic resonance imaging. Independent component analysis of voxelwise thalamic functional connectivity maps parcellated the cortex into thalamus-related networks, and thalamic subdivisions associated with these networks were delineated. Functional connectivity of (1) networks with the thalamus and (2) thalamic subdivision seeds were examined. In patients, functional connectivity of the salience network with the thalamus was decreased and localized to the ventrolateral (VL) and ventroposterior (VP) thalamus, while that of a network comprising the cerebellum, temporal and parietal regions was increased and localized to the mediodorsal (MD) thalamus. In patients, thalamic subdivision encompassing the VL and VP thalamus demonstrated hypoconnectivity and that encompassing the MD and pulvinar regions demonstrated hyperconnectivity. Our results extend the implications of disrupted thalamocortical networks involving specific thalamic nuclei to dysfunctional large-scale brain network dynamics in schizophrenia pathophysiology.

## Introduction

Accumulating evidence suggests the role of the thalamus and its associated cortical connectivity in the pathophysiology of schizophrenia. Large-scale meta-analyses of brain structure^1,2^, function^3,4^, and chemistry^5,6^ studies have reported various defects of the thalamus in patients with schizophrenia and suggest this brain region as one of most affected in the disorder. The thalamus, however, is comprised of cytoarchitecturally distinct nuclei^7^. Parallel pathways link specific thalamic nuclei to disparate cortical areas and comprise topographically organized thalamocortical networks that support a wide array of functions spanning sensory, motor, cognitive and affective domains^7,8^. Incorporating the multinuclear structure of the thalamus and its distinct pattern of projections to cortical regions, several disease models have postulated the diversity of symptoms observed in schizophrenia to be characterized by the differential involvement of specific thalamocortical networks^9–11^.

In vivo examination of specific thalamocortical network disturbances in schizophrenia became possible with the advent of connectivity-based segmentation methods that delineate subdivisions of the thalamus based on their pattern of cortical connectivity^12,13^. Conventionally, the cortex is parcellated into large cortical regions of interest (ROIs) that broadly correspond to the primary targets of specific thalamic nuclei, as detailed in animal studies. Each thalamic voxel is then singularly associated with one of these anatomically defined ROIs to which it is most strongly connected, and specific thalamocortical networks are localized within the resultant thalamic subdivisions that resemble thalamic nuclei identified from histological studies^14,15^. In the schizophrenia literature, diffusion tensor imaging (DTI) and resting-state functional magnetic resonance imaging (fMRI) studies employing these methods have reported consistent patterns of thalamocortical network abnormalities involving the mediodorsal (MD) nuclei in association with decreased connectivity between frontal ROIs and the thalamus, as well as the ventrolateral (VL) and ventroposterior (VP) nuclei with increased connectivity between motor- and sensory-related ROIs and the thalamus in chronic^10,16–18^ and early psychosis patients^17,19^. The comparable topography of anatomical and functional connectivity abnormalities reported in the literature, however, also highlights limitations of conventional connectivity-based segmentation approaches.

Although connectional anatomy constrains functional connectivity, the two measures are not isomorphic^20^, and brain regions can demonstrate functional connectivity in the absence of direct structural linkages via polysynaptic connections, common-source connections, and other configurations of bidirectional circuits^21–24^. The conventional use of cortical ROIs that are anatomically defined based on the projections of thalamic nuclei, however, has confined previous examinations of thalamocortical functional connectivity to white matter pathways. Furthermore, spatially distinct regions that are not necessarily anatomically bound comprise intrinsically coherent large-scale networks fundamental to brain functional organization^21–24^. Contributions of these intrinsic networks in thalamocortical functional connectivity have been suggested by the spatial nonuniformity of correlations observed within cortical ROIs^15^, but they have been insufficiently accounted for. Thus, in prior investigations, anatomically defined ROIs may have served as functionally inaccurate ROIs^25^ that render topography of thalamocortical functional connectivity and its abnormalities much simplified and constricted to anatomical pathways. Overcoming these limitations carries significance beyond methodological imperatives in the schizophrenia literature when considering the extensive reports of dysfunctional large-scale brain network dynamics in patients across illness stages^26–29^.

Increasing efforts investigating the role of the thalamus in human cortical organization have advanced functional perspectives in connectivity-based segmentation or parcellation approaches. Their application in psychosis, however, remains to be examined. In recent studies in healthy subjects, intrinsic brain networks have been utilized as functionally defined ROIs and delineated functional subdivisions of the thalamus that, unlike their anatomical counterparts, span across major nuclei groups and exhibit distributive connectivity with multiple cortical regions and networks^30,31^. Furthermore, characterizing the distributive connectivity of thalamic nuclei, or subregions, has allowed insights into the role of the thalamus as a critical integrative node that mediates brain network dynamics. In this context, incorporating large-scale brain networks in the examination of thalamocortical functional connectivity abnormalities implicated in schizophrenia pathophysiology could not only augment the details of specific thalamocortical network disruptions with an advanced analytical approach but also provide further insight into the mechanisms underlying dysfunctional large-scale brain network dynamics.

To do so, the present study adopted a data-driven method^31^ delineating thalamus-related functional networks and comprehensively examined their connectivity abnormalities in patients with first-episode psychosis (FEP). We employed this sample to minimize the effects of potentially degenerating processes associated with the chronic course of the disorder, such as the effects of medication, poor diet, less exercise, or heavy smoking. Independent component analysis (ICA) was performed on the whole-brain voxelwise functional connectivity maps calculated for each voxel within the thalamus. The resultant independent components (ICs), which we refer to as “network ROIs,” comprised a set of spatially distinct brain regions that were functionally connected to common seed voxels within the thalamus across subjects and parcellated the cortex into a set of thalamus-related functional networks. Functional subdivisions of the thalamus were delineated by localizing the thalamic seed voxels associated with the network ROIs using regression models. Unlike the conventional ‘winner takes all’’ strategy that assigns only singular associations between ROIs and thalamic voxels, this approach allowed associations with multiple networks. The distributive functional connectivity of thalamic nuclei, or subregions, with various networks would be evident in the spatial overlaps among subdivisions. In the primary analysis, functional connectivity of the network ROIs with the thalamus was examined. This was comparable to previous connectivity-based segmentation studies, in that specific thalamocortical networks were localized within the thalamic subdivisions. In the secondary seed-based analysis, functional connectivity of the thalamic subdivisions with the rest of the brain was examined.

## Results

The demographic and clinical characteristics of the FEP subjects and HCs are summarized in Table 1, and their group differences were examined with chi-square analyses and independent t tests for categorical and continuous data, respectively, using IBM SPSS version 24 (IBM, Chicago, IL, USA). The two groups significantly differed in years of education (*t* = − 2.07, *p* < 0.05), which was missing for one FEP subject, and IQ (*t* = − 4.17, *p* < 0.01).

**Table 1:** Demographic and clinical variables of the subjects.

	FEP	HC	Statistical analysis^a^	
	(n = 40)	(n = 40)	*X*^2^ or *T*	*p*
Age (years)	22.88 ± 5.64	22.58 ± 3.94	0.28	0.78
Sex (male/female)	18/22	20/20	0.20	0.65
Handedness (right/left)	35/5	35/5	0	1.00
Education (years)	13.26 ± 2.01	14.15 ± 1.83	− 2.07	0.04
IQ	98.67 ± 13.51	111.90 ± 14.68	− 4.17	0.00
Duration of illness (months)	5.74 ± 3.82			
Medication^b^ (mg)	9.73 ± 7.61			
**PANSS**				
Total	69.03 ± 14.26			
Positive symptoms	16.48 ± 4.86			
Negative symptoms	17.45 ± 5.65			
General symptoms	35.10 ± 7.62			
GAF	46.25 ± 10.00			

Data are presented as the means ± standard deviations (SD).

Abbreviations: *FEP* first-episode psychosis, *HC* healthy control, *PANSS* Positive and Negative Syndrome Scale, *GAF* Global Assessment of Functioning.

^a^Independent t test or Welch’s t test if the variances were not equal, χ^2^ analysis or Fisher’s exact test for categorical data.

^b^Olanzapine-equivalent dose.

**Figure 1:**
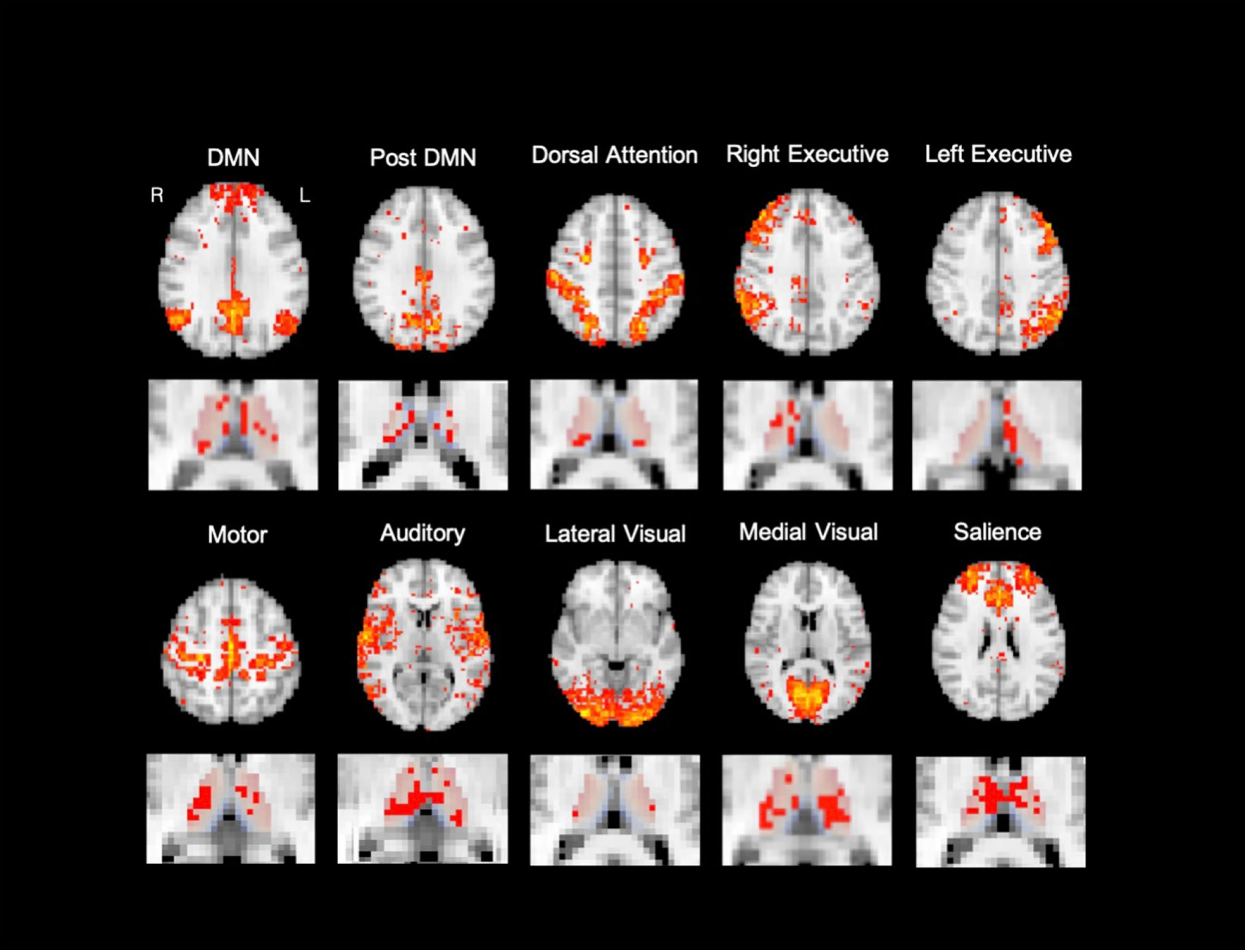
Thalamus-related cortical networks identified from ICA and the associated thalamic subdivisions for 10 representative cortical networks.

ICA resulted in 20 thalamus-related network ROIs, and thalamic voxels associated with each network ROI were localized using regression models to delineate functional subdivisions of the thalamus. The resultant ICs included cortical networks resembling the generally observed resting-state networks (RSNs)^32,33^, as well as subcortical and cerebellar components (Fig. 1 and Figure S1). The resultant thalamic subdivisions broadly corresponded with thalamic mappings of functional brain networks previously reported^31,34^, and overlaps among subdivisions were also evident (Fig. 1 and Figure S1). The results of ICA and regression analyses most resembling the 10 networks reported by Yuan et al.^31^ are presented in Fig. 1. These networks corresponded to the default mode (DMN), posterior DMN, left and right executive (frontoparietal), auditory, dorsal attention, motor, salience, lateral visual, and medial visual networks.

**Figure 2:**
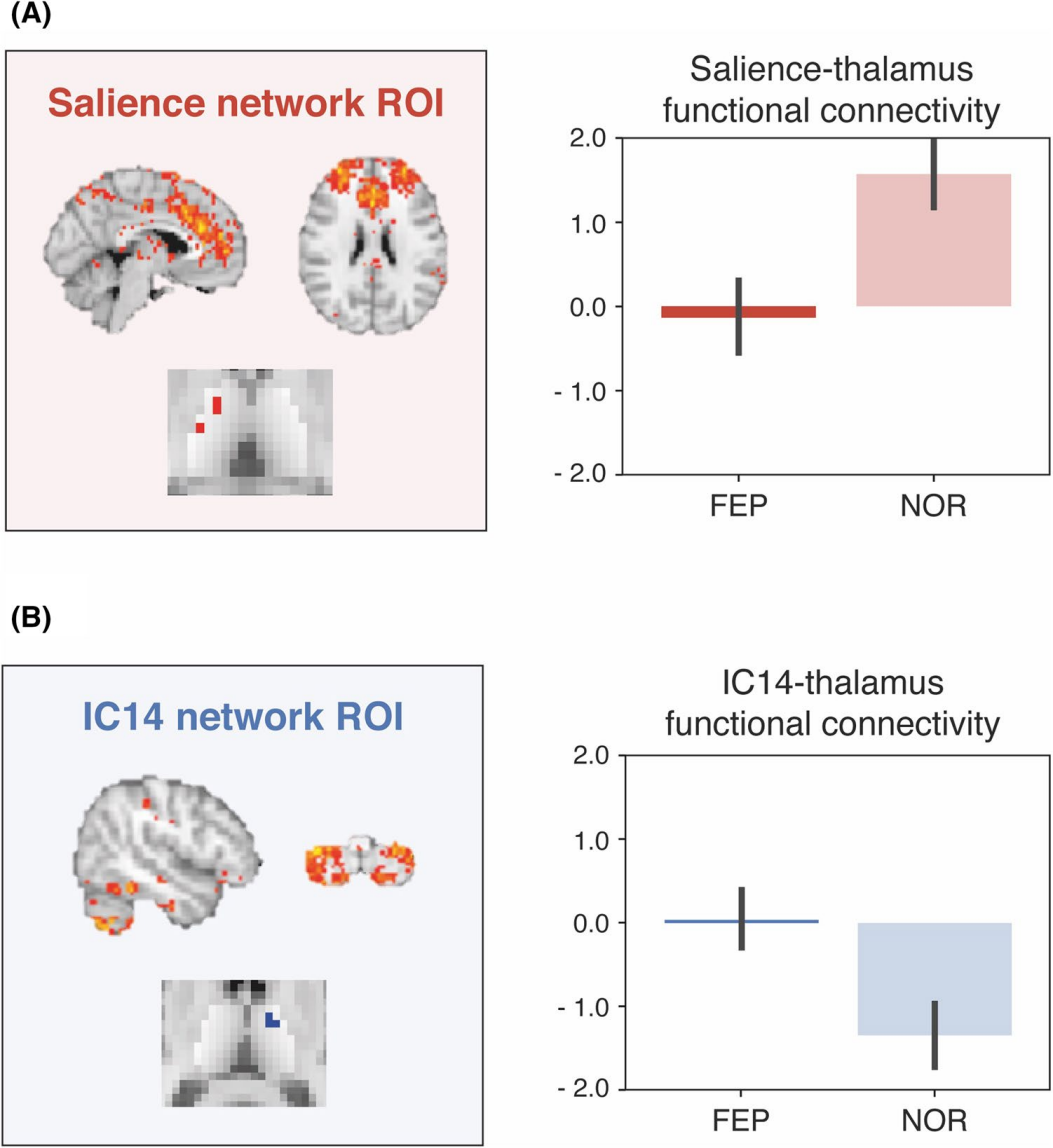
Altered functional connectivity of the network ROIs with the thalamus in the FEP patients. Red foci on the thalamus localize where patients exhibited reduced connectivity with the network ROI; blue foci on the thalamus localize where patients exhibited stronger connectivity with the network ROI. Bar graphs represent mean functional connectivity values.

Our primary analysis examining functional connectivity of the network ROIs and the thalamus revealed decreased salience network-thalamus connectivity, which localized to thalamic clusters encompassing the right VL and VP nuclei, in the FEP group (Fig. 2). The FEP group also exhibited increased functional connectivity of the IC14 network, which comprised the cerebellum, temporal and parietal regions, and the thalamus that localized to clusters encompassing the left MD nucleus (Fig. 2).

**Figure 3:**
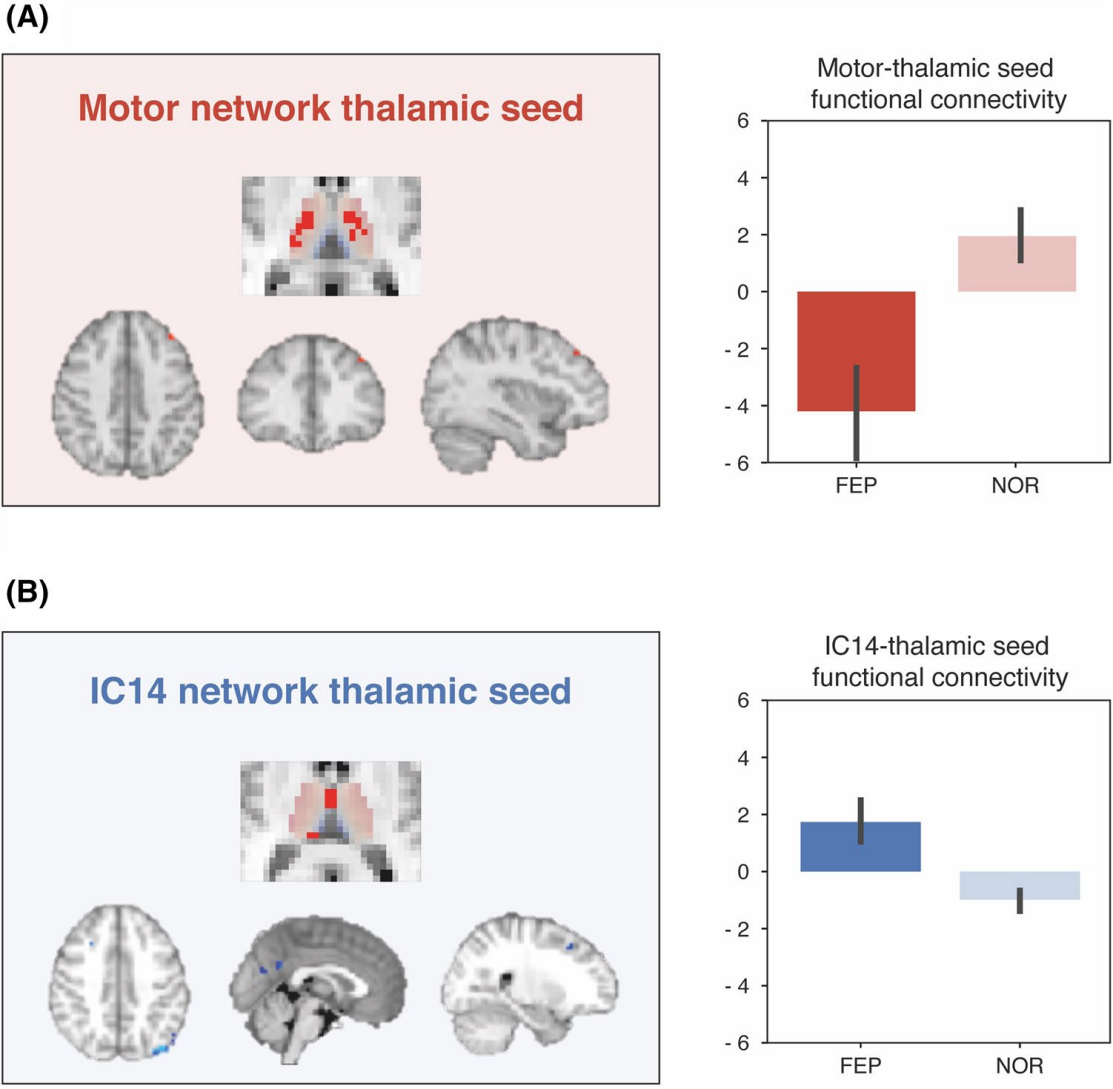
Dysconnectivity of the thalamic subdivisions in the FEP patients revealed in the seed-based analyses. Red foci on the cortex mark areas where patients exhibited reduced thalamic coupling with the thalamic seed; blue foci on the cortex mark areas where patients exhibited stronger coupling with the thalamic seed. Bar graphs represent mean functional connectivity values.

The subsequent seed-based analysis examined functional connectivity of thalamic subdivision seeds with the rest of the brain. In the FEP group, the thalamic subdivision encompassing the VL and VP aspects demonstrated hypoconnectivity with a cluster in the left middle frontal gyrus (Fig. 3). This thalamic seed was delineated by its functional connectivity with the motor network. The thalamic subdivision encompassing the MD and pulvinar regions of the thalamus demonstrated hyperconnectivity with clusters in the left lateral occipital, precuneus, posterior cingulate, and right middle frontal regions of the cortex (Fig. 3). This thalamic seed was delineated by its functional connectivity with the IC14 network ROI that demonstrated increased functional connectivity with the MD thalamus in the FEP group.

## Discussion

The present study aimed to incorporate intrinsic brain networks into the examination of thalamocortical functional connectivity abnormalities implicated in the pathophysiology of FEP. To do so, abnormalities in patients with FEP were comprehensively studied using thalamus-related functional network ROIs and their corresponding thalamic subdivisions. Our primary analysis revealed abnormal functional connectivity of the network ROIs with the thalamus in the FEP group. Decreased connectivity of the salience network localized to the VL and VP aspects of the thalamus and the increased connectivity of the IC14 network, which comprised the cerebellum, temporal and parietal regions, localized to the MD thalamus. Seed-based analysis of thalamic subdivisions further demonstrated thalamocortical dysconnectivity in the FEP group. The motor-thalamic subdivision, which encompassed ventral aspects of the thalamus, exhibited hypoconnectivity, and the IC14-thalamic subdivision, which encompassed MD and pulvinar regions, exhibited hyperconnectivity. Our results provide further support for and extend the contexts of disrupted functional interactions involving specific thalamocortical networks in psychosis.

The salience network, which exhibited decreased functional connectivity with the thalamus in patients with FEP, plays an integral role in mediating the switch between default mode and task-related states of brain connectivity^35^. Dysfunctions involving this network in facilitating the engagement of executive frontoparietal networks and disengaging the DMN upon cognitive demands have been postulated to be a core pathology of schizophrenia in the triple-network model^36,37^. Considering the role of the thalamus as an integrative hub^30,31^, abnormal salience network-thalamus functional connectivity may extend thalamic involvement to aberrant triple-network dynamics. Thalamic nuclei, or subregions, exhibit extensive functional connectivity with multiple large-scale networks and demonstrate a pattern similar to cortical connector hubs that, likewise, may support the integration of information across and mediate interactions among networks^30^. In our analysis, such distributive connectivity was evident in the considerable overlaps among functional subdivisions of the thalamus. Overlaps observed in the MD and pulvinar regions of thalamic subdivisions associated with the DMN and executive frontoparietal networks have suggested possible contributions of these higher-order nuclei in mediating interactions between these two networks that are frequently reported as competitive^38–40^. Similarly, overlaps among thalamic subdivisions associated with the salience network, DMN and executive frontoparietal networks could suggest thalamic involvement in triple-network dynamics. In the present and previous^31,34^ analyses, these overlaps were observed in the anterior, ventral, and pulvinar nuclei.

Functional connectivity abnormalities involving the IC14 network in our analyses highlight the involvement of higher-order thalamic nuclei. In the FEP group, increased IC14-thalamus connectivity localized to the MD thalamus, and the IC14-thalamic subdivision seed, which localized to the MD and pulvinar regions of the thalamus, exhibited hyperconnectivity. Postmortem studies reporting alterations in volumes^41,42^ and neuronal numbers^43,44^ in these higher-order nuclei provided early evidence for the differential involvement of specific thalamic nuclei in schizophrenia^45^. Thalamocortical models of schizophrenia have also long posited the centrality of these higher-order nuclei in tandem with cognitive impairments^46–49^. While these disease models are supported by animal and lesion studies that implicate thalamocortical network abnormalities involving these higher-order nuclei in mechanisms of cognitive impairments^50–54^, reports of cognitive, as well as clinical, correlates in patient studies remain scarce and mixed^55^. Interpretations of our present results are also limited by the lack of cognitive measures and this network’s resemblance to typical RSNs.

The MD and ventral thalamus highlighted in our results are consistent with the literature. The direction of change and cortical regions associated with functional connectivity abnormalities involving these nuclei, however, are incongruous. In prior thalamocortical investigations in psychosis, abnormal functional interactions of the MD thalamus have been reported in relation to decreased connectivity with frontal regions^17,55^ and the ventral thalamus in relation to increased connectivity with motor/sensory regions^10,17,55^. Discrepancies regarding our results probe differences in the composition of specific thalamocortical networks that stem from differences in ROI definitions, as well as in strategies localizing the thalamic voxels associated with specific thalamocortical networks. While a direct comparison of the present and previous results goes beyond the scope of this study, we further elaborate on the aptness and complexities entailed in our methodological approach, which aimed to delineate the topography of thalamocortical networks that better reflect brain functional organization. Correspondence between our network ROIs parcellated using a data-driven approach and cortical ROIs defined a priori with anatomical basis, as well as between thalamic subdivisions delineated in association with respective ROIs, are low, as they should be. Discrepancies, however, may also stem from functional obscurities of specific thalamocortical networks delineated using conventional cortical ROIs. The anatomical basis of these ROIs is not the only issue. These ROIs have also been coarsely defined in that they encompass extended regions of gray matter and comprise functionally heterogeneous areas, which can also have differential connectivity profiles with the thalamus^56–59^. Additionally, the conventional use of the “winner takes all” strategy averages the signals of these ROIs. Furthermore, the use of such coarsely defined cortical ROIs has ipso facto precluded spatial specificity in the cortex and the rest of the brain. Two previous connectivity-based segmentation studies, one functional and the other anatomical, employed seed-based analyses of a complementary nature to address this limitation^17,60^. Interestingly, whereas the anatomical connectivity of the PFC-thalamic subdivision seed was found to be confined to the PFC cortical ROI of the seed^16^, its functional connectivity extended beyond its PFC ROI and was found to be distributive with regions of the prefrontal-cingulo-parietal “executive control” network^17,61^ and suggested that the functional topography of thalamocortical networks involves large-scale brain networks.

Results of our seed-based analysis conducted with functional subdivisions of the thalamus, on the other hand, highlight the complexities of our methods. This analysis revealed hyperconnectivity of the seed encompassing the higher-order MD and pulvinar nuclei with small clusters of the lateral and parietal occipital, posterior cingulate, precentral, and frontal regions and hypoconnectivity of the seed encompassing the ventral thalamus with a small frontal cluster. The distributive functional connectivity of these thalamic subregions with extensive cortical networks may, at least partially, explain this perplexing pattern of results. While considerable overlaps among thalamic subdivisions corroborated the role of the thalamus as a critical integrative hub for brain functional networks, this pattern may have also complicated between-group statistical inferences by blurring the direction of and cortical regions associated with thalamocortical functional abnormalities observed in patients. For instance, the subdivision demonstrating hyperconnectivity encompassed the MD and pulvinar regions, which were localized in association with the IC14 network. However, both the MD and pulvinar regions were also observed, as well as previously reported^31,34^, to be associated with the DMN, executive frontoparietal, salience, and medial visual networks, and the pulvinar was additionally associated with lateral visual, auditory and dorsal attention networks. The subdivision demonstrating hypoconnectivity encompassed the ventral lateral and posterior thalamus and was localized in association with the motor network. However, these thalamic subregions also functionally interacted with the executive, salience, and medial visual networks.

Several other limitations of the present study merit consideration. First, many of the patients with FEP were on antipsychotics at the time of the scan. Although the effects of medication would be relatively small compared with those in patients with chronic schizophrenia, antipsychotics and antidepressants have subtle but measurable impacts on generalized and specific brain tissues^62,63^. In regard to the quality of our fMRI data, its limited spatial resolution, which was also resampled for the computational demands of analysis, makes the full representation of the complex heterogeneity of thalamic nuclei difficult. We could only approximately locate the major thalamic nuclei using the Talairach thalamic nuclei labels within the MNI template. The nonisotropic voxel shape of our EPI image is another possible limitation. Suggestions for further methodological refinements should also be considered. First, our analysis utilized network ROIs that represented large-scale patterns of thalamocortical functional connectivity. While these thalamus-related functional networks bore resemblance to typical RSNs generally identified based on large-scale corticocortical functional connectivity^32,33,64^, methodologically integrating these corticocortical networks in the analysis of thalamocortical networks could help better facilitate implications of RSNs or graph theoretical measures in probing dysfunctional brain dynamics in schizophrenia. Second, our network ROIs were identified by applying data-driven ICA on the dataset that was combined across the FEP and HC subjects to allow operation on a larger sample size. By doing so, however, abnormalities present in the patient data may have affected the initial data reduction and may have subsequently fed into stages leading to the inference of group differences. Nonetheless, our ICs displayed similar spatial patterns with those previously reported in healthy samples^31^ and supported the validity of our method. A follow-up study in a larger sample that would allow the examination of abnormalities in patients using ICA results from the study’s healthy subjects could help clarify this issue. Last, issues inherent to ICA should also be addressed^65^. A main concern is the dependency on the choice of dimensionality. ICs are known to show degrees of variability with changes in the number of ICs specified. It has been reported that low dimensionality generally results in components representing “networks” of regions with related activity, while increasing the dimensionality acts to divide those regions across multiple components^66–68^. Although extracting a larger number of ICs may present a finer network structure^69,70^, it is also possible that increasing model order results in less reliable ICs and a decrease in ICA repeatability, which is another inherent problem of the iterative algorithm^71^. Employing an unsupervised clustering approach and evaluating the stability of diverse segmentation results to obtain an optimal solution of the number of thalamic subdivisions^34^ may offer an alternative approach to ICA.

In conclusion, the present study examined thalamocortical functional connectivity abnormalities in patients with FEP in the context of large-scale brain networks involving the thalamus. Our results not only provide further support for disrupted thalamocortical networks involving the MD and ventral thalamus but also extend the implications of thalamocortical abnormalities to dysfunctional large-scale brain network dynamics.

## Methods

### Participants

Forty patients with FEP were recruited from the inpatient and outpatient clinics at the Seoul National University Hospital (SNUH) between April 2010 and March 2016. The inclusion criteria for FEP subjects were being between the ages of 16 and 40, satisfying the diagnosis of a schizophreniform disorder, schizophrenia or schizoaffective disorder as assessed via the Structured Clinical Interview for the Diagnostic and Statistical Manual of Mental Disorders, Fourth Edition (DSM-IV) Axis I Disorders (SCID-I), and having a duration of illness that was less than two years. The Positive and Negative Syndrome Scale (PANSS) and the Global Assessment of Functioning (GAF) were administered to assess psychotic symptoms and the functional status of patients with FEP. At the time of scanning, 33 patients were receiving antipsychotics. The average daily dose of antipsychotics in an olanzapine-equivalent dose is presented in Table 1. Additionally, 5 patients were taking antidepressants, and 19 patients were taking anxiolytics. Course specifiers were available for 39 of the 40 patients, 21 of whom were in acute episode and 18 in partial remission. Forty age- and sex-matched healthy control (HC) subjects were recruited via Internet advertisement. The exclusion criteria for HCs included having a past or current diagnosis of an Axis I disorder as assessed via Structured Clinical Interview for DSM-IV Axis I Disorders Non-patient Edition (SCID-NP) and having any first- to third-degree biological relatives with psychotic disorder. The common exclusion criteria for all participants included substance abuse or dependence (except nicotine), neurological disease or significant head trauma, medical illness that could accompany psychiatric symptoms, and intellectual disability (intelligent quotient [IQ] < 70). IQ was estimated by administering the Korean version of the Wechsler Adult Intelligence Scale (K-WAIS). All participants received a thorough explanation of the study procedure and provided informed written consent. This study was conducted in accordance with the Declaration of Helsinki and was approved by the Institutional Review Board of SNUH.

### Neuroimaging data acquisition

All imaging data were acquired using a Siemens 3 T Magnetom Trio Tim Syngo MR B17 scanner with a 12-channel head coil. T1-weighted images (T1) were obtained for each participant to enable spatial localization and normalization and were acquired using the three-dimensional (3D) magnetization-prepared rapid-acquisition gradient echo (MPRAGE) sequence in the sagittal plane with the following parameters: repetition time (TR) = 1670 ms, echo time (TE) = 1.89 ms, voxel size = 1mm^3^, field of view (FOV) = 250 mm, flip angle = 9° and 208 slices. Resting-state fMRI data were acquired using echo-planar imaging (EPI) in the axial plane with the following parameters: TR = 3500 ms, TE = 30 ms, FOV = 240 mm, flip angle = 90°, matrix = 128 × 128, voxel size = 1.9 × 1.9 × 3.5 mm^3^, 35 slices, and 116 timepoints. During the fMRI scan, which lasted for 6 min and 58 s, each subject was instructed to relax with his/her eyes closed but not to fall asleep.

To minimize possible head movements and subsequent motion artifacts, the subjects were asked to move as little as possible during image acquisition, and head cushions were also used. All images were visually inspected to exclude the presence of artifacts or gross anatomical abnormalities that could impact image processing.

### Data preprocessing

All preprocessing steps were conducted using FMRIB Software Library (FSL) version 6.0. Preprocessing for functional data included discarding the first 4 echo-planar images, motion correction, slice timing correction, automated brain extraction, and highpass temporal filtering (> 0.01 Hz). Next, the motion parameters and signals from white matter and cerebral spinal fluid (CSF) were removed from each voxel by linear regression. The resulting data were then registered to the standard 2-mm isotropic Montreal Neurological Institute (MNI) space template by linearly registering the EPI data to the brain-extracted T1 and then applying the transform resulting from the T1 to MNI brain nonlinear registration to the registered EPI data. Finally, the data were resampled to a 3 × 3 × 3 mm^3^ voxel size. No spatial smoothing was performed.

### Functional delineation of thalamocortical networks

**Figure 4:**
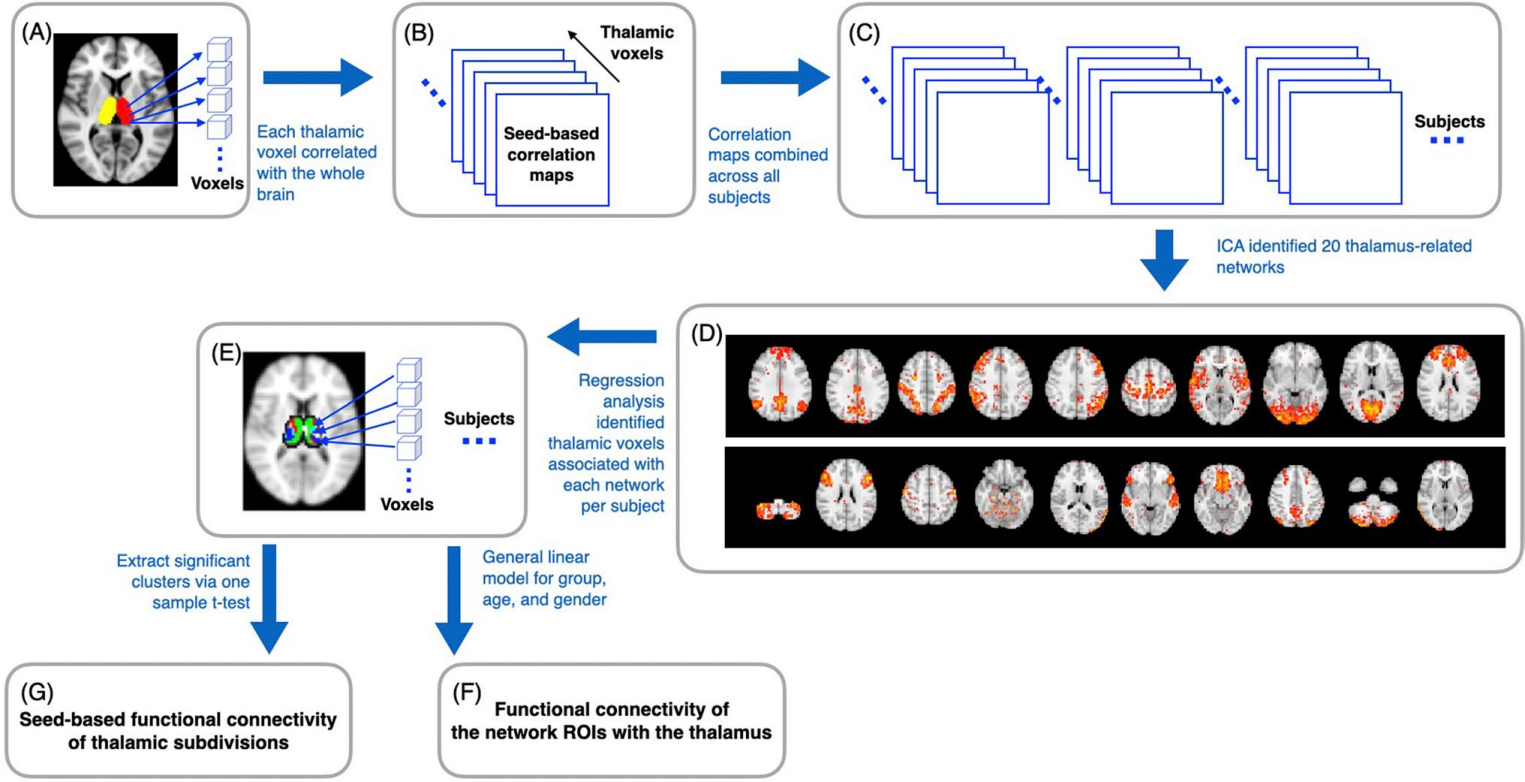
Schematic flow of the thalamocortical functional connectivity analysis.

We adopted a method proposed by Yuan et al.^31^ to identify large-scale thalamus-related functional networks and to delineate the associated functional subdivisions of the thalamus. The analysis steps were as follows (Fig. 4).

First, the thalamus mask was obtained by combining the left and right thalamus from the Harvard–Oxford subcortical atlas and resampling into 3 × 3 × 3 mm^3^ in MNI space. This mask included 606 voxels. The functional connectivity of each thalamic voxel to all voxels across the whole brain was calculated using Pearson’s correlation coefficient, resulting in 606 seed-based correlation maps for each subject. These maps were Fisher-transformed to Z-scores and concatenated into a single 4D dataset, which was used as the input for spatial ICA. The shape of the final 4D dataset was 60 × 72 × 60 × 48,480 voxels, since a single volume had 60 × 72 × 60 voxels, and there were 606 volumes for each of the 80 subjects.

Spatial ICA was performed using FSL’s MELODIC (Multivariate Exploratory Linear Optimized Decomposition into Independent Components)^72^ and decomposed the 4D data into 20 spatially independent components (ICs). The number of components was determined with reference to recent studies^31,73^. The resulting ICs, which are referred to as “network ROIs,” represent intrinsically coherent functional networks that originate from common regions within the thalamus across subjects.

To delineate the functional subdivisions of the thalamus, thalamic voxels associated with each network ROI were localized in two steps^74,75^. First, their temporal dynamics were extracted using the group-level spatial maps of ICs as a set of spatial regressors in a general linear model (GLM). Following, the subject-specific spatial maps of the thalamic subregions were found using the extracted timecourses as a set of temporal regressors in a GLM. The resultant individual maps of thalamic subdivisions were used in the subsequent thalamocortical functional connectivity analyses, and their corresponding major thalamic nuclei were identified using the Talairach thalamic nuclei labels within the MNI template.

### Thalamocortical connectivity

The primary analysis examined the functional connectivity of each network ROI with the thalamus. The individual maps of thalamic subdivisions resulting from regression analyses were concatenated across subjects, and between-group differences were tested for significance using FSL randomise^76^, with age and sex modeled as covariates. Statistical maps were thresholded at the cluster level and considered significant at corrected *p* < 0.05.

The subsequent seed-based analysis examined functional connectivity of the thalamic subdivisions with the rest of the brain. Seeds were extracted from the individual maps of thalamic subdivisions resulting from regression analyses via voxelwise one sample *t* tests across subjects using FSL randomise, with threshold-free cluster enhancement (TFCE) and *p* < 0.05 as the statistical threshold^77^. The mean time series was extracted from each seed for each subject and entered into a general linear model. Between-group functional connectivity differences were tested for significance using FSL randomise, with age and sex modeled as covariates, and TFCE using *p* < 0.05 as the statistical threshold.

Both analyses were performed separately for every thalamic subdivision. Talairach thalamic labels were used to identify the thalamic nuclei that corresponded to the significant clusters resulting from our primary analysis and seed extraction.

## Supplementary Information

Supplementary Figure S1.

Supplementary Figure S2.
