# Supplementary figures and images for: Mapping thalamocortical functional connectivity with large-scale brain networks in patients with first-episode psychosis
Source: Sci Rep. 2021 Oct 6;11:19815. doi: 10.1038/s41598-021-99170-7 (PMC8494789; doi:10.1038/s41598-021-99170-7)

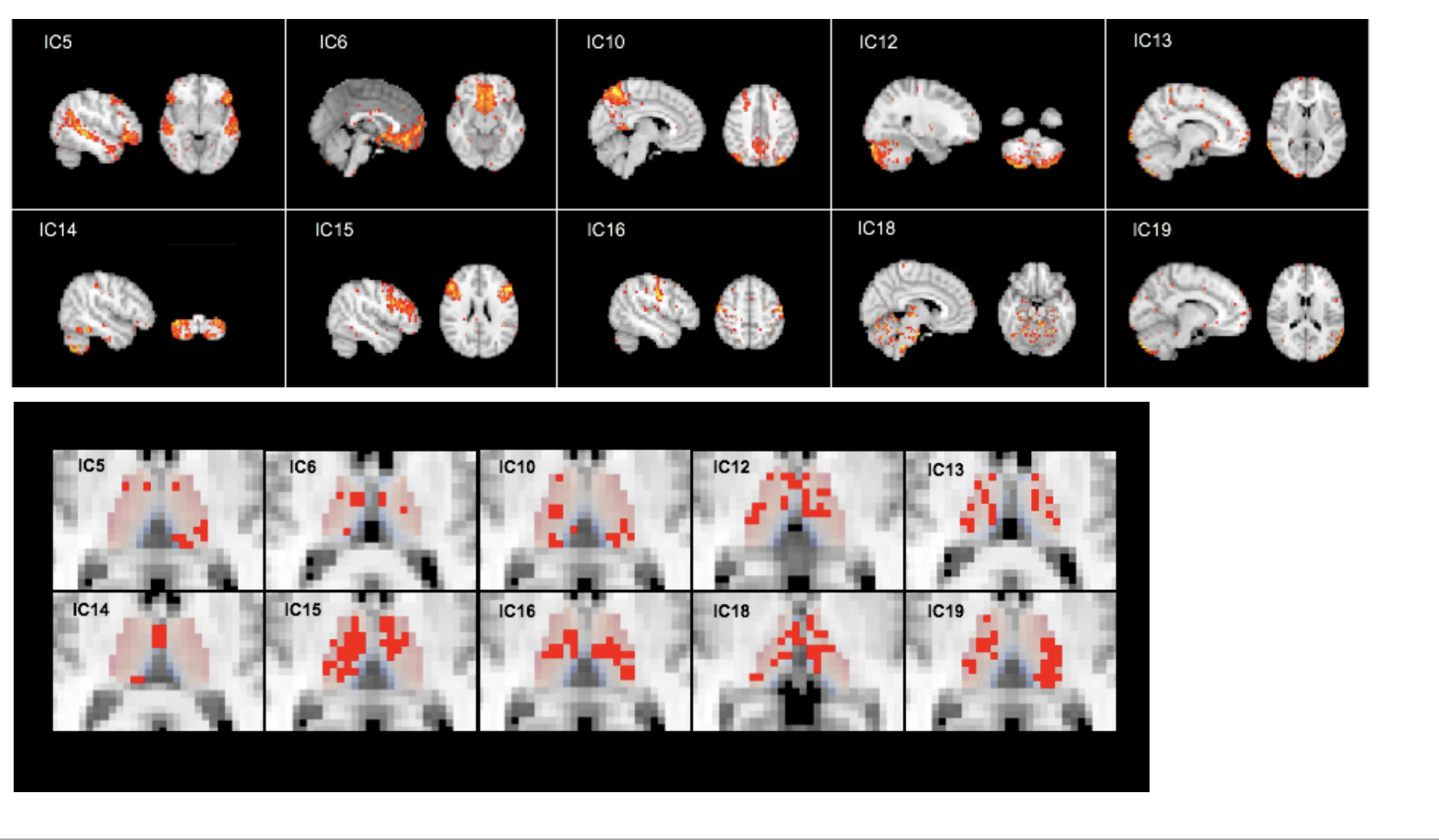

Supplement: Supplementary file 1 — Supplementary Figure S1. [file 41598_2021_99170_MOESM1_ESM.tiff]
